# “Because we all have to grow up”: supporting adolescents in Uganda to develop core competencies to transition towards managing their HIV more independently

**DOI:** 10.1002/jia2.25552

**Published:** 2020-08-31

**Authors:** Chloe Lanyon, Janet Seeley, Stella Namukwaya, Victor Musiime, Sara Paparini, Helen Nakyambadde, Christine Matama, Anna Turkova, Sarah Bernays

**Affiliations:** ^1^ School of Public Health University of Sydney Sydney NSW Australia; ^2^ Department of Global Health and Development London School of Hygiene and Tropical Medicine London United Kingdom; ^3^ MRC/UVRI and LSHTM Uganda Research Unit Entebbe Uganda; ^4^ Africa Health Research Institute (AHRI) Durban South Africa; ^5^ Department of Paediatrics and Child Health Makerere University Kampala Uganda; ^6^ Research Department Joint Clinical Research Centre Kampala Uganda; ^7^ Nuffield Department of Primary Care Health Sciences University of Oxford Oxford United Kingdom; ^8^ Research Department Joint Clinical Research Centre Kampala Uganda; ^9^ Clinical Trials Unit University College London London United Kingdom

**Keywords:** adolescents, transition, paediatric, adherence, psychosocial support, viral suppression, HIV

## Abstract

**Introduction:**

Sustaining optimal adherence is the major challenge facing adolescents living with HIV (ALHIV), particularly in low‐resource settings, where “second‐line” is often the last accessible treatment option. We explored the knowledge and skills adolescents need in order to maintain improved adherence behaviours, and the specific ways clinicians and caregivers may support young people to do so more independently.

**Methods:**

We conducted individual, in‐depth interviews with 20 ALHIV aged 10 to 18 years in Uganda in 2017 to 2018. All participants had recently commenced second‐line treatment as part of a clinical trial. We used thematic qualitative analysis to examine adherence experiences and challenges while on first‐line therapy, as well as specific supports necessary to optimise treatment‐taking longer‐term.

**Results:**

Adherence difficulties are exacerbated by relatively rapid shifts from caregiver‐led approaches during childhood, to an expectation of autonomous treatment‐taking with onset of adolescence. For many participants this shift compounded their ongoing struggles managing physical side effects and poor treatment literacy. Switching to second‐line typically prompted reversion back to supervised adherence, with positive impacts on self‐reported adherence in the immediate term. However, this measure is unlikely to be sustainable for caregivers due to significant caregiver burden (as on first line), and provided little opportunity for clinicians to guide and develop young people’s capacity to successfully adopt responsibility for their own treatment‐taking.

**Conclusions:**

As ALHIV in sub‐Saharan Africa are attributed increasing responsibility for treatment adherence and HIV management, they must be equipped with the core knowledge and skills required for successful, self‐directed care. Young people need to be relationally supported to develop necessary “*adherence competencies*” within the supportive framework of a gradual “transition” period. Clinic conversations during this period should be adolescent‐focussed and collaborative, and treatment‐taking strategies situated within the context of their lived environments and support networks, to facilitate sustained adherence. The disclosure of adherence difficulties must be encouraged so that issues can be identified and addressed prior to treatment failure.

## 1 INTRODUCTION

With vast improvements in survival resulting from effective prevention of mother‐to‐child transmission (PMTCT) and antiretroviral treatment (ART) for HIV in high‐burden, resource‐stretched countries, adolescence has emerged as a critical priority area for HIV care [1, 2, 3]. Limited improvements in survival have been observed among this age‐group relative to their paediatric and adult counterparts [3, 4], and morbidity rates among adolescents in sub‐Saharan Africa are not declining at the same pace as other age groups [3]. As for other chronic conditions, provision of HIV care during adolescence is characterised by unique management challenges as major cognitive, psychosocial, physical and sexual developmental changes take place [5, 6, 7, 8]. This is illustrated by relatively poorer HIV‐care outcomes with high rates of attrition from clinical care services [9, 10, 11] and compromised ART adherence [10, 12, 13]. Accompanied by exploration of risk‐associated sexual behaviours [14, 15], this has far‐reaching consequences for individuals and public health outcomes alike [16, 17].

Importantly, adolescence involves the shift of responsibility for HIV care, from caregiver to adolescent [18]. To support the development of mechanisms which enable independent self‐management of treatment compliance and health literacy among adolescents, there is a need to characterise the process of successful transition among adolescents, including development of evidence‐based approaches to guide clinicians [10, 19]. In high‐burden, resource‐stretched settings, the transition to adult care may not be demarcated by a discrete movement between paediatric and adult facilities as occurs in developed settings [20, 21, 22, 23]. Thus, contextually appropriate approaches to formalising this process are needed to facilitate intentional adolescent capacity‐development and treatment continuity [9, 24].

In this paper, we conceptualise “transition” as being the shift young people make away from caregiver‐driven (or “mediated”) to increasingly autonomous, self‐directed treatment‐taking and HIV care; accompanying the broader, contemporaneous developmental transition toward independence. Our applied approach is grounded in the social‐ecological theory for transition preparedness [25, 26]; incorporating both individual elements of transition preparedness and consideration of the influential role of the surrounding physical and social environments and support systems [25, 27]. We investigated the challenges encountered by ALHIV during the adolescent transition period, particularly those related to treatment‐taking, and identified core competencies required to manage their health independently. And secondly, the specific ways in which clinicians and caregivers may equip ALHIV with the knowledge and skills required for successful transition to “adult” HIV care.

## 2 METHODS

We present the results of a qualitative study conducted among 20 ALHIV (aged 10 to 18 years) attending a leading HIV clinic in Kampala, Uganda. The clinic is well‐resourced, and offers separate paediatric and adult care. Transfer to adult care typically occurs once a young person is in their early twenties. This study was conducted over a 12‐month time period: 2017 to 2018.

The study is a qualitative sub‐study of the ongoing “ODYSSEY” trial (Once‐daily DTG based ART in Young people versus Standard thErapY) (NCT NCT02259127) [28]. The clinical trial is an open‐label, randomised, non‐inferiority, basket trial evaluating the efficacy and safety of DTG plus 2 nucleos(t)ides (NRTIs) versus standard of care in HIV‐positive adolescents age <18 years starting first‐line ART (ODYSSEY A) or switching to second‐line (ODYSSEY B).

We recruited ALHIV who had experienced treatment failure (ODYSSEY B), as identified by high viral load or morbidity. These participants were presumed to be encountering adherence challenges and had recently commenced second‐line treatment through the clinical trial. All clinical trial participants who were eligible to take part in the qualitative study were invited to do so, with the final five participants recruited purposively to ensure a representative balance of age and gender across the sample.

Data were collected using in‐depth, semi‐structured interviews, which were conducted by trained local researchers in a private room within the paediatric clinic (caregivers were not present). The interview guide was developed based on knowledge from both the existing literature and the authors’ previous research, but primarily focused on generating detailed accounts of individual experiences of treatment‐taking within its broader context. It was then revised and adapted in light of emerging analytical areas of interest; including experiences of growing up with HIV; ART‐adherence; expectations of adulthood and “adult” models of care; management of relationships and onward disclosure. We present findings from the first wave of interviews, in which participants reflected on their treatment experiences on first‐line therapy, prior to switching.

All participants were aware of their HIV status prior to recruitment. Appropriate national and institutional ethics approvals were obtained, specifically, from the London School of Hygiene and Tropical Medicine and the Joint Clinical Research Centre, Uganda. Participant consent or assent were obtained, and caregiver consent obtained for assenting participants aged younger than 18 years.

Audio‐recorded interview data were transcribed verbatim and then translated into English for analysis. Participant identifying details were removed and pseudonyms applied. Transcript coding was done individually (manually) by the first and last author. These initial codes were discussed and reconciled into a Codebook. The Codebook was reviewed by the second and third author, who were familiar with the data. A thematic qualitative analysis was conducted, with emerging themes discussed between the authors to ensure the integrity of inductive data interpretation (including understanding of nuances specific to the local context). Deviant case analyses were conducted to attain representative depth of analysis.

## 3 RESULTS

Table 1 presents participant characteristics. The experiences of participants reflected a range of diverse situations which shaped their capacity to adhere to their HIV treatment. However, all participants had recently been moved onto second‐line ART, and all reported experiencing some challenges maintaining optimal adherence.

**Table 1:** Participant characteristics (n = 20)

Sex	12 male, 8 female
Median age (age range)	14.5 years (10 to 18 years)
Mode of HIV infection	All participants had vertically acquired HIV
Antiretroviral therapy	All participants were receiving second‐line ART therapy
Living situation/caregiver/s	13 were living with parent/s and 7 were living with wider family
Education and employment	17 were attending school, 2 were technical apprentices, 1 was employed
Clinical care centre	All were receiving care from the same paediatric HIV clinic

ART, antiretroviral therapy.

### 3.1 Individual‐level (adolescent) considerations

#### 3.1.1 Evolving models of care

Our findings suggest adolescents are attributed more responsibility for their treatment‐taking with increasing age, with dwindling involvement from their caregivers. Although participants anticipated becoming “fully responsible” for treatment‐taking “when [they] grow older” (George, male, age 10), we argue this expectation often faltered in reality as they were not adequately equipped with necessary accompanying knowledge and strategies to successfully navigate and sustain the complex demands of transition to self‐care.

For several of our participants, the time period during which they had assumed responsibility for their own treatment‐taking predated them being disclosed to regarding their diagnosis and understanding the necessity of consistent treatment‐taking [29, 30]. Dennis, male, age 14, shared how this knowledge deficit proved problematic for him:

“There were some problems, because there came a situation when I didn’t know why I was taking this medicine, they took long to tell me… I was not taking them because I didn’t know what they were for so I asked myself why I had to since I was young… I used to ask my mother many times but she would just say that you must take them… So when I stopped, on coming here the viral load went high to about 2000, and we went to the counsellor and they asked if I knew, and it was my mum to tell me… Before I knew that I had HIV it was difficult because I just didn’t understand why I had to yet others were not taking.”

#### 3.1.2 Knowledge deficits as a barrier to self‐management

Aligned with study inclusion criteria, all participants were aware of their HIV‐positive status. At the time of data collection, however, HIV and related treatment literacy remained variable and frequently limited. Participants described garnering HIV‐related information from non‐specialist sources such as radio, television and overheard conversations between laypeople, and tended to absorb overly simplistic, blunt and often distressing messages. Zack male, age 15, recalls his reaction upon learning of his diagnosis in this way:

“I got so scared… I knew that I was going to die. *Why did you think that you were going to die?* They used to say that HIV kills. *Where did you hear this?* Over the radio and even amongst people.”

Relevant information is typically provided in clinic visits. However, this appeared not to have been understood and integrated into the young people’s knowledge. Thus, ambiguous explanations given during childhood often persisted unamended [31] rather than increasing in sophistication with maturity. Sophia, female, age 17, shared simply, “I was born with HIV.” Some appeared unaware of their sizeable knowledge gaps and did not know to enquire. When prompted, however, many participants had specific questions related to their illness which had not yet been voiced. Representative of this issue, Denna, male, age 11, disclosed:

*“Do you perhaps have any questions about your illness?* Yes, I have always wanted to know where HIV came from. *Have you ever asked your mother about this?* Sometimes she is too busy so I get scared to ask. *What about at the clinic?* I fear the health care workers also. *What do you think will happen if you asked them?* I don’t know but I fear and sometimes I forget.”

Generally, understanding of specific administration requirements for treatment taking, such as appropriate timing, were hazy. When asked the time he was meant to take his medication, Mark, male, age 12, responded, “any time that I want.” Jasmine, female, aged 17, shared a different concern:

“Sometimes I would find myself late and I would give up because I know that day is wasted… They told us that we can only take the medication at most five minutes passed the scheduled time.”

Others were aware that they needed to take treatment every day, but struggled due to side‐effects such as “headache and nausea” (Zack, male, age 15), presuming these to be unavoidable.

#### 3.1.3 Individual adherence strategies

At the time of being interviewed, all knew why they were taking treatment, and the majority had grasped that adherence was important. Sam, male, age 12, shared, “I want to take my medication for my survival.” Similarly frightened by the prospect of switching, Denna, male, age 11, resolved he would “make an extra effort to take medication at all the times [he was] supposed to take them.” However, renewed commitment in the absence of additional support may be less likely to succeed. A handful of participants had devised individual strategies to support adherence. Teddy, female, age 14, and Ethan, male, age 18, set alarms to prompt them to take their treatment; Lazarus, male, age 18, took his at the time of a particular television programme, and Mariam, female, age 14, upon Muslim call to prayer. Jasmine, female, age 17, initiated changing the time of administration to facilitate adherence:

“I would wake up late but I had to go to school and yet I had not yet showered, so before I know it is getting to 7am yet I have not yet taken my medication, so I decided to change it.”

The development and use of these individual adherence strategies indicates a willingness and evolving capacity to self‐care, however, these self‐driven, apparently autonomous approaches were exceptional; and the need for additional support is evidenced by their need to transition to second‐line therapy despite their individual efforts.

### 3.2 Interpersonal‐level considerations (caregivers, schools, workplaces)

#### 3.2.1 Caregiver‐mediated treatment‐taking

During childhood and early adolescence, capacity for self‐care exists within the framework of caregiver‐dependence and support. Dan, male, age 17, shared; “they (grandparents) are the ones who give me the money for transport to come to the clinic, they also ensure that I have juice or milk to take with my medication.” In later adolescence, some participants continued to benefit from caregiver support in contingency management. Sheena, female, age 17, explained; “if I don’t feel well, I let her (mother) know and she finds a solution.” Extended family and siblings assisted with providing support for several participants. In other accounts, however, caregivers were unsure how to respond to concerns and crisis situations arising in the home environment. Their capacity to respond appeared to be determined by the extent to which they had been equipped by healthcare workers with accessible resources and strategies.

#### 3.2.2 Transfer, rather than transition, of responsibility

Caregivers frequently struggled to juggle work and caring commitments. Denna, male, age 11, shared his impression that his mother was “too busy” to respond to questions about his illness. Ethan, male, age 18, similarly explained his grandmother “is usually busy, so she may not know that I take my medicine.” Given these limitations, transfer of responsibility for treatment‐taking may have necessarily occurred in an unplanned, abrupt manner, rather than being implemented gradually; leaving young people with unreliable treatment supporters, or for some, “no people to remind or to help them” (Sophia, female, age 17).

Being moved to second‐line treatment prompted more intensive adherence support, as Bob, male, age 15, recalls: “because I was missing, they (caregivers) started to give me my medication.” While reverting to caregiver‐mediated treatment taking may be initially helpful, this approach fails to address underlying causes of non‐adherence, and previous difficulties encountered by caregivers are likely to resurface; making this unlikely to be a sustainable model of support.

#### 3.2.3 Caregiver‐mediated competency development

A handful of participants shared strategies implemented by their caregivers which developed their ability to self‐manage treatment‐taking, evolving to a model of “supporting” rather than “delivering” treatment. For example, Zack, male, aged 15, reported; “I take my medication at the same time with mother, so if one of us has forgotten we remind each other.” Dan, male, aged 17, shared; “I keep my medicine in their (grandparents) bedroom on a table, so in the evenings I go with my milk to their bedroom then I show them [that I] take the medicine.” In both scenarios, the adolescent is encouraged to initiate taking treatment on time, but within a protective net of indirect caregiver supervision. Other caregivers provided psychological support for their children with notable benefit. Jasmine, female, age 17, shared:

“I told him that I was fed up of taking the medication every day, but he (father) told me that I have to take my medication, and I insisted and told him that I was fed up, then he encouraged me.”

#### 3.2.4 Role of schools and workplaces

The benefit of collaboration with schools (and for older adolescents, workplaces) also proved to be an essential aspect of navigating impediments to treatment. For example, by approving absences necessary to attend HIV clinic appointments, providing education (“they taught us about HIV in class;” Sheena, female, age 17), combatting stigma when teachers “treat us (HIV‐positive children) just like the other children” (Teddy, female, age 14), and discreetly storing and distributing ART medication.

We found that maintaining the confidentiality of HIV status was a universal priority, in light of significant concerns about bullying and stigma, and yet many participants lacked strategies to manage their treatment‐taking discreetly in their school, workplace and (for some) HIV‐discordant home environments. This issue created critical difficulties within boarding school contexts (in the absence of necessary support) as Saidat, female, age 16, recalls:

“When I was in boarding school, I would be afraid to remove my medication to take it in the presence of the other students… because they will make fun of me or feel disgusted by me, like they do with the other children.”

Lazarus, male, age 18, also avoided taking medication to work because “the pills were too big, as soon as someone else saw them they would instantly know… that I am sick.” Not always verbalised, these issues persisted unacknowledged; and in Saidat’s particular case, resulted in a period of compromised adherence totalling “three years.”

### 3.3 Organization‐level considerations (providers, clinicians)

#### 3.3.1 Adolescent‐centred clinical interactions

The role of clinicians is central to the facilitation of transitions towards inter‐dependent self‐management of treatment adherence, however, we observed occasions where the structure of clinical interactions posed a significant barrier to their potential effectiveness. We found that caregivers rather than adolescents were often positioned at the centre of consultations, and that this had not necessarily evolved as they aged (particularly for those who had attended the clinic since childhood). Mariam, female, age 14, described a recent appointment where she was not addressed directly by the clinician:

*“*Sometimes (the counsellor) speaks to me, but many times she speaks to Mummy. *Today what did you speak about with the counsellor?* She was writing in the file and Mummy was answering her.”

Inadvertent exclusion of adolescents from clinical interactions appeared to limit the development of their health literacy and ability to self‐care, and emerged as being particularly problematic when caregiver support was withdrawn. Despite these challenges, enthusiasm to engage with clinicians and in health education persisted. Teddy, female, age 14, shared her (yet unmet) desire to learn more about HIV; “I like coming here because I am keen to know about my health. *Do they tell you about your health when you come here?* No they do not tell me, but I would like to know.”

#### 3.3.2 Clinician‐mediated competency development

It is revealing that participants were very responsive to instances when clinicians had initiated discussion and provided counselling. Jasmine, female, age 17, demonstrated how fostering disclosure of adherence difficulties can lead to their timely resolution, such as through joint problem solving;

“I told a health care worker that I take my medication in the morning at 7am and when I come back from school at 6pm… but I told her that time is sometimes challenging for me. The counsellor told me that I could choose a time that was more convenient for me, and from that time I started to choose the time when to take my medication myself.”

In an environment where disclosure of HIV‐positive status remains complex and challenging in the community setting, clinicians may be the only contacts other than the primary caregiver or immediate family to be aware of an adolescent’s HIV‐positive status. Sheena, female, age 17, shared:

“I want to tell people what it is like for me taking the medicine, I would like them to encourage me. Mainly things to do with my treatment… I would like to speak to a friend.”

Clinicians have a potentially vital role to play as key supporters of psychological as well as physical wellbeing; central to holistic care and adolescent resilience. Teddy, female, age 14, shared the benefit she received by partaking in the clinical trial: “the study helps encourage us to take our medication well, and not feel sad and isolate ourselves.”

## 4 DISCUSSION

In accordance with the wider chronic disease literature describing best practice management during adolescence, our findings indicate that a deliberate “transitional” period in HIV care is required to facilitate successful transfer of HIV‐management responsibility, from caregiver to adolescent [7, 22, 23, 32]. We suggest adopting a social‐ecological approach to the evolving model of care provision during this life‐stage [25], incorporating both individual elements of transition preparedness, and harnessing the influential role of surrounding physical and social environments and available support systems [25, 27].

Individuals must be equipped with core competencies required to engage with, and manage, the transition to autonomous treatment‐taking and HIV care; for example, HIV literacy, self‐efficacy and skill‐acquisition, beliefs, goals and expectations, and psychosocial wellbeing [25, 27]. The nature and implications of their diagnosis, rationale and window of opportunity to take treatment daily, and strategies to manage missed doses and medication side‐effects are essential to adherence counselling. For this purpose, established “transition checklists” may provide a valuable tool to broadly establish the extent of individual transition preparedness and guide ongoing care (where adapted, contextually suitable checklists are available [33, 34]). We suggest that HIV‐counselling content increase in sophistication pre‐emptively with evolving maturity [35, 36, 37]. Ultimately, the optimal timing and rate of adolescent transition should be tailored to the neurocognitive developmental status of the individual, based on longitudinal assessment and contemporaneous understanding of individual capacity [25, 38], rather than chronological age [39].

As young people mature, the relational influence of caregivers (who typically determine the success of HIV care during childhood) remains integral. However, the onset of adolescence should herald the onset of a deliberate, developmentally appropriate transition period which increasingly supports adolescents to partner with their treating clinicians directly [23, 40]. Schools and workplaces may facilitate the transition process by providing supportive environments; in particular, by offering logistical assistance such as discreet treatment storage and medical leave.

This approach emphasizes that effective care during the transitional adolescent period entails a joint approach, which actively involves clinician, caregiver and adolescent collaboratively in the therapeutic process [7, 25, 38]. Aligned with the core foundation of social‐ecological theory as pioneered by Bronfenbrenner [41], the adolescent should be positioned at the centre of the transition framework, and more specifically, at the centre of clinical interactions. Such encounters should foster an environment of uninhibited discussion, collaboration and support [11, 29, 40, 42]. We found that treatment regimens were far more likely to be successful when they were developed in consultation with individual young people themselves [6, 21, 23, 43]. This may be achieved by eliciting and seriously considering the priorities of ALHIV, such as preserving secrecy of their HIV‐positive status, engaging in education and employment opportunities, establishing peer and intimate relationships, and maintaining realistic hope for the future [42, 44, 45, 46, 47]. Treatment plans should be adapted to contextual factors [6, 8, 23], such as by tailoring scheduled times for treatment‐taking to prevent conflict with school and work commitments. In this way, regimen tolerability may be optimised, adherence incentivized and barriers to adherence pre‐emptively addressed [39, 48, 49]. Similarly, when adherence is compromised, rather than conceptualising a young person as having “failed” in this regard (which may limit disclosure of future adherence challenges [29, 48]), they should be re‐framed as being the expert on their own individual circumstances [48, 50]. Thereby, encouraging the adolescent to engage with caregivers and clinicians alike to identify their specific support needs [13, 37, 39, 48]. Provision of training for healthcare professionals in the delivery of adolescent‐centred care is likely to convey benefit to HIV‐care outcomes by optimising the efficacy of therapeutic encounters [9, 21, 23, 40]. Clinical interventions should be paired with strategic engagement of caregivers and relevant stakeholders (including teachers and employers) to foster supportive environments; integral to adolescent success during the transition period.

### 4.1 Strengths and limitations

Previous qualitative research conducted among children and adolescents in similar settings has demonstrated their tendency to ration the candour with which they speak about adherence challenges, due to a desire to protect themselves and their relationships with others; given the moralized nature of discussions surrounding adherence [29, 31, 42]. A significant strength of this study is the recruitment of participants who had recently transitioned to second‐line therapy. This provided us with a unique opportunity to discuss adherence challenges as being in the past, after they had been acknowledged; thereby facilitating greater transparency in participants’ accounts.

A potential limitation of this study is that the clinic in which it was conducted is a relatively well‐resourced speciality HIV centre, as such, our findings risk over‐estimating the resources available in smaller, more remote settings. Conscious of this, we have focussed on transition as primarily taking place in community settings (homes and schools) with clinician guidance provided only during scheduled clinic appointments; an approach which should be replicable.

## 5 CONCLUSIONS

As ALHIV are attributed greater responsibility for their treatment‐taking and management, they must be equipped with the essential knowledge and capabilities required. Our findings suggest improved HIV outcomes in high‐burden, resource‐stretched settings may be achieved through the implementation of an intentional “transition period” in HIV care, incrementally supporting adolescents toward sustainable, autonomous management. We suggest adopting a social‐ecological approach to transition care, whereby ALHIV are relationally supported in clinic, home, school and workplace environments to develop necessary “*adherence competencies;*” specifically, through ongoing dialogue about HIV treatment literacy; routine management; identification of contingency strategies should social or physical environmental factors impede treatment taking; and disclosure of adherence difficulties as standard practice (allowing barriers to be addressed early). The proposed approach acknowledges the evolving relationship between adolescent, caregiver and clinician, and fosters efficacious collaboration in relation to HIV care throughout the dynamic transition process.

## COMPETING INTEREST

The authors have no competing interest to declare.

## AUTHORS’ CONTRIBUTIONS

SB and JS designed the study which forms the basis of the data presented in this manuscript, with the support of SP and VM. SN collected the data with the supervision and support of SB and JS. VM contributed to the overview of data collection with JS and SB. Data analysis and drafting of the manuscript was done by CL and SB with support provided by JS, SN and SP. HN and CM contributed to data management and early contributions to data analysis. AT and VM contributed to the development of the manuscript. All authors read, reviewed and approved the final manuscript.

## Supporting information


**Appendix S1.** ODYSSEY TRIAL TEAM*
